# Effect of repetition on the behavioral and neuronal responses to ambiguous Necker cube images

**DOI:** 10.1038/s41598-021-82688-1

**Published:** 2021-02-10

**Authors:** Vladimir Maksimenko, Alexander Kuc, Nikita Frolov, Semen Kurkin, Alexander Hramov

**Affiliations:** 1 Neuroscience and Cognitive Technology Laboratory, Center for Technologies in Robotics and Mechatronics Component, Innopolis University, 1 Universitetskaya str., Innopolis, Republic of Tatarstan Russia 420500; 2 Saratov State Medical University, 112 Bolshaya Kazachia str., Saratov, Russia 410012

**Keywords:** Cognitive control, Perception, Decision, Sensory processing

## Abstract

A repeated presentation of an item facilitates its subsequent detection or identification, a phenomenon of *priming*. Priming may involve different types of memory and attention and affects neural activity in various brain regions. Here we instructed participants to report on the orientation of repeatedly presented Necker cubes with high (HA) and low (LA) ambiguity. Manipulating the contrast of internal edges, we varied the ambiguity and orientation of the cube. We tested how both the repeated orientation (referred to as a stimulus factor) and the repeated ambiguity (referred to as a top-down factor) modulated neuronal and behavioral response. On the behavioral level, we observed higher speed and correctness of the response to the HA stimulus following the HA stimulus and a faster response to the right-oriented LA stimulus following the right-oriented stimulus. On the neuronal level, the prestimulus theta-band power grew for the repeated HA stimulus, indicating activation of the neural networks related to attention and uncertainty processing. The repeated HA stimulus enhanced hippocampal activation after stimulus onset. The right-oriented LA stimulus following the right-oriented stimulus enhanced activity in the precuneus and the left frontal gyri before the behavioral response. During the repeated HA stimulus processing, enhanced hippocampal activation may evidence retrieving information to disambiguate the stimulus and define its orientation. Increased activation of the precuneus and the left prefrontal cortex before responding to the right-oriented LA stimulus following the right-oriented stimulus may indicate a match between their orientations. Finally, we observed increased hippocampal activation after responding to the stimuli, reflecting the encoding stimulus features in memory. In line with the large body of works relating the hippocampal activity with episodic memory, we suppose that this type of memory may subserve the priming effect during the repeated presentation of ambiguous images.

## Introduction

Perception and memory are the essential brain functions that help us interact with each other and the environment. Perception reflects the identification and interpretation of sensory evidence to understand the presented information. Memory stands for the faculty by which the brain encodes sensory or other information, stores, and retrieves it when needed. Memory influences our perceptual abilities so that we recognize better the objects we have seen before^1^. A growing body of literature examines this effect in experimental paradigms, including the repeatedly presented sensory stimuli. They provide substantial experimental evidence that repeated presentation of an item facilitates its subsequent detection or identification, a phenomenon known as *priming*^2^. This effect also persists when reexperiencing particular features, such as color or shape, but not the stimulus as a whole. For instance, in a visual search task, if the target color on the present trial is red, as it was on the previous trial, a search is facilitated, whereas the green target color slows the search^1^.

Literature refers to these effects as object-based and feature-based priming^3^. There is a view that feature-based priming engages an implicit memory, whereas object-based priming relies on the explicit memory^4^. Implicit memory allows people to perform specific tasks without conscious awareness of these previous experiences. In contrast, explicit memory refers to the conscious, intentional recollection of previous experiences and concepts. Probably, these types of priming also relate to the bottom-up and top-down components of attention. The bottom-up component refers to involuntary attentional guidance to salient stimuli because of their inherent properties relative to the background. The top-down component refers to the internal guidance of attention based on prior knowledge and current goals^5^. According to Kristjansson et al., the stimuli and the circumstances of the task in each case dictate what sort of priming occurs^3^.

Thus, priming occurs at many different levels of the perceptual hierarchy ranging from lower (feature-based) to higher (object-based) levels, depending on the stimulus, task, and context. Therefore, it appears to modulate neuronal activity in different brain regions. Neuroimaging studies reported a deceased fMRI signal for primed vs. unprimed stimuli in various brain regions, a phenomenon called *repetition suppression*. The repetition suppression in posterior regions evidenced priming of perceptual processes. The effect in more anterior (prefrontal) regions accompanied priming of conceptual processes^6^. In addition to repetition suppression, repeated stimulus presentation can also enhance brain responses relative to its first presentation, a phenomenon called *repetition enhancement*. While the repetition enhancement usually occurs in the brain regions associated with explicit memory, reflecting incidental conscious memory for the previous encounter with a stimulus, it has occasionally been reported in the brain regions associated with priming, showing repetition suppression under other conditions^7^.

Here we analyzed how repetition affected the behavioral and neuronal responses to ambiguous visual stimuli, Necker cubes. We instructed subjects to respond to the cube’s orientation (left or right). Manipulating the contrast of internal edges, we varied the ambiguity and orientation of the cube. We assumed that the brain treated the contrast of separate edges on the low processing levels and defined the orientation at the higher levels. The Necker cubes with low (LA) and high (HA) ambiguity had almost the same morphology, and we assumed their similar processing on low levels^8^. We also supposed that responding to HA stimulus orientation required more information; therefore, HA processing lasted longer, causing greater neuronal response on higher levels. Finally, we hypothesized that high ambiguity made the subjects to rely on the top-down processes, such as expectations and memory^9^. Thus, we tested how both the repeated orientation (stimulus factor) and the repeated ambiguity (top-down factor) modulated neuronal and behavioral response.

On the behavioral level, we observed higher speed and correctness of the response to the HA stimulus following the HA stimulus and a faster response to the right-oriented LA stimulus following the right-oriented stimulus.

The prestimulus theta-band power grew when the HA stimulus followed the HA stimulus. The source analysis revealed an increased activation of the right and the left insula, right frontal cortex, and the left postcentral cortex. It may indicate activation of the neural networks related to attention and uncertainty processing before HA stimulus onset. The repeated HA stimulus enhanced activity in the thalamus, hippocampus, and the left parietal and postcentral gyri for 0.1–0.35 s post-stimulus onset. The right-oriented LA stimulus following the right-oriented stimulus enhanced activity in the precuneus and the left inferior frontal gyri for $\sim 0.3$ s before the behavioral response. Together, these results may suggest that stimulus processing relies on the matching information between the current and previously presented stimuli. Increased hippocampal activation during the earlier (0.1–0.3 s) stage of HA stimulus processing may evidence retrieving information to disambiguate it and define the orientation. Increased activation of the precuneus and the left prefrontal cortex before responding to the right-oriented LA stimulus following the right-oriented stimulus may indicate a match between the orientations at the higher processing levels. Finally, we observed an increased hippocampal activation for $\sim 1.5$ s after responding to the HA stimuli and the right-oriented stimuli. It may reflect the encoding of the percept-related and the decision-related stimulus features in memory after its offset.

Our finding coincides with a very recent work of Kim et al.^10^ manifesting that neural repetition suppression of neuron-level activity in the hippocampus accompanies enhanced EEG power at low frequencies. In line with the large body of works relating the hippocampal activity with episodic memory, we suppose that this type of memory may subserve the priming effect during the repeated presentation of ambiguous images.

**Figure 1:**
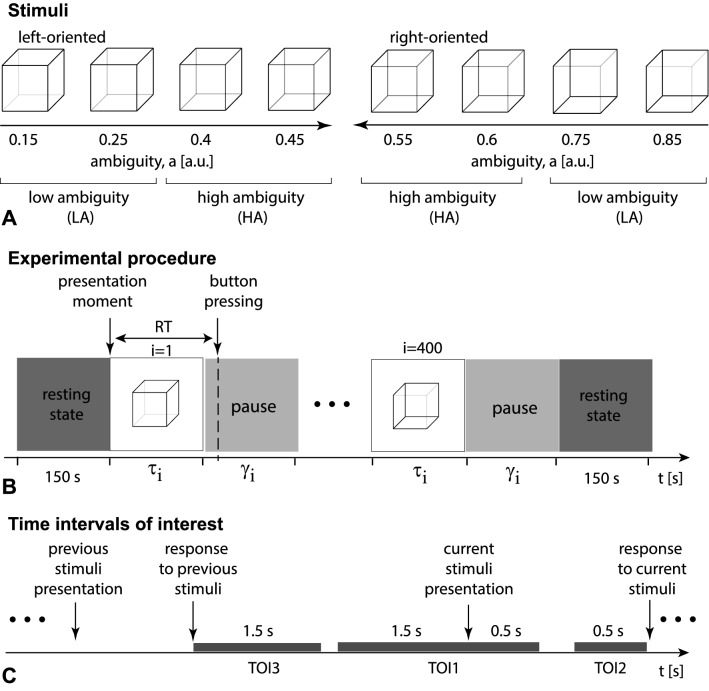
The set of visual stimuli includes Necker cubes with different orientation and ambiguity (**A**). The experimental procedure (**B**) contains 400 stimuli presentations alternating with abstract image presentations; RT defines the subject’s response time. (**C**) The time intervals of interest, TOI: TOI1—a 2 s interval time-locked to the current stimulus onset including 1.5 s prestimulus and 0.5 s post-stimulus segments; TOI2—a 0.5 s interval preceded the behavioral response to the current stimulus; TOI3—a 1.5 s interval followed the behavioral response to the previously presented stimulus.

## Methods

### Participants

Twenty healthy subjects (9 females) aged 26–35 with normal or corrected-to-normal visual acuity participated in the experiments. All of them provided written informed consent in advance. All participants were familiar with the experimental task and did not participate in similar experiments in the last six months. The experimental studies were performed under the Declaration of Helsinki and approved by the local Research Ethics Committee of the Innopolis University.

### Experimental procedure

We chose an ambiguous drawing of a Necker cube as a bistable visual stimulus ^11–13^. The ambiguity, as well as the orientation of the cube’s image, was determined by the balance between the brightness of the inner edges forming a left-lower ($b_l=1-a$) and right-upper ($b_r=a$) squares, where $a\in [0,1]$ was a normalized edge’s luminance in a gray-scale palette. Thus, the limit cases of $a=0$ and $a=1$ corresponded to unambiguous 2D projections of left- and right-oriented cubes, respectively, whereas $a=0.5$ determined a completely ambiguous cube’s image.

In our experiment, we used a set of the Necker cube images with $a=\{0.15,0.25,0.4,0.45,0.55,0.6,0.75,0.85\}$ (Fig. 1A). On the one hand, this set could be separated into subsets of left-oriented $a=\{0.15,0.25,0.4,0.45\}$ and right-oriented cubes $a=\{0.55,0.6,0.75,0.85\}$. On the other hand, in accordance with our previous study^14^, this set could be also divided into low-ambiguous (LA) images $a=\{0.15,0.25,0.75,0.85\}$, which are easily interpreted by an observer, and high-ambiguous (HA) images $a=\{0.40,0.45,0.55,0.60\}$, whose interpretations requires more effort.

We demonstrated 14.2 cm Necker cubes on a white background using a $24^\prime \prime$ monitor with the $1920\times 1080$ pixels resolution and 60 Hz refresh rate. The distance between the subject and the monitor was 0.7–0.8 m, and a visual angle was $\sim 0.25$ rad. The whole experiment lasted around 40 min for each participant, including short recordings of the eyes-open resting EEG state ($\approx 150$ s) before and after the main part of the experiment. During experimental sessions, the cubes with predefined values of *a* (chosen from the set in Fig. 1A) were randomly demonstrated 400 times, each cube with a particular ambiguity was presented about 50 times.

We randomized parameter *a* in the following way. First, we formed a vector *A*(1...400), including all images (50 images for each value of *a*). Then, we randomized indexes in this vector by using the function *randperm* in MATLAB. It returned a row vector containing a random permutation of the indexes from 1 to 400 without repeating elements. Finally, this randomized vector of indexes determined the order of stimuli presentation. We randomized time of the stimuli presentations and pauses between them as $t_{min} + rand*(t_{max} - t_{min})$. Here, $t_{max}$ and $t_{min}$ defined minimal and maximal presentation/pause time, and *rand* is a MATLAB function that returns a single uniformly distributed random number in the interval (0,1).

Each *i*-th stimulus exhibition lasted for a time interval of $\tau$ and varied from $\tau _{min}=1$ s to $\tau _{max}=1.5$ s. Pauses, $\gamma$ between the subsequent presentations of the Necker cube images contained the abstract picture exhibition and varied from $\gamma _{min}=3$ s to $\gamma _{max}=5$ s (Fig. 1B). We instructed participants to press either the left or right key, responding to the left or the right stimulus orientation. For each stimulus, we estimated a behavioral response by measuring the response time, RT, which corresponded to the time passed from the stimulus presentation to button pressing (Fig. 1B).

### EEG recording and processing

The EEG signals were recorded using the monopolar registration method and the classical extended 10–10 electrode scheme. We recorded 31 signals with two reference electrodes A1 and A2 on the earlobes and a ground electrode N just above the forehead. The signals were acquired via the cup adhesive Ag/AgCl electrodes placed on the “Tien–20” paste (Weaver and Company, Colorado, USA). Immediately before the experiments started, we performed all necessary procedures to increase skin conductivity and reduce its resistance using the abrasive “NuPrep” gel (Weaver and Company, Colorado, USA). After the electrodes were installed, the impedance was monitored throughout the experiments. Usually, the impedance values varied within a 2–5 k$\Omega$ interval. The electroencephalograph “Encephalan-EEG-19/26” (Medicom MTD company, Taganrog, Russian Federation) with multiple EEG channels and a two-button input device (keypad) was used for amplification and analog-to-digital conversion of the EEG signals. This device possessed the registration certificate of the Federal Service for Supervision in Health Care No. FCP 2007/00124 of 07.11.2014 and the European Certificate CE 538571 of the British Standards Institute (BSI). The raw EEG signals were filtered by a band-pass FIR filter with cut-off points at 1 Hz (HP) and 100 Hz (LP) and by a 50-Hz notch filter by embedded a hardware-software data acquisition complex. Eyes blinking and heartbeat artifact removal was performed by Independent Component Analysis (ICA) using EEGLAB software^15^.

We divided preprocessed EEG signals into trials. We considered two trials for each stimulus: the first trial had a length of 4 s, and its middle point was time-locked to the stimulus presentation. The second one also had a 4 s length, but its middle point was time-locked to the behavioral response (when the subject pressed the button). We calculated wavelet power (WP) in the frequency band of 4–40 Hz using the Morlet wavelet for each trial. The number of cycles, *n* depended on the signal frequency, *f*, as $n=f$. To minimize between-subject variability, we considered normalized wavelet power (NWP) by contrasting WP on all trials to the WP averaged over 40-s baseline EEG before the experiment: $\mathrm {NWP}=(\mathrm {WP}-\mathrm {WP}_{baseline})/\mathrm {WP}_{baseline}$. All calculations were performed using the Fieldtrip toolbox in MATLAB. After the EEG preprocessing procedure, we excluded some trials due to the remaining high-amplitude artifacts. As a result, the number of trials varied from 170 to 296 (M=227, SD=42) in a group of subjects.

Unlike the other works on ambiguous stimuli processing^16,17^, we did not present completely ambiguous stimuli, such as a fully symmetrical cube with $a=0.5$. Moreover, we instructed subjects to be as correct as possible. Therefore, we supposed that the subjects responded on the cube orientation based on the acquired sensory information, rather than the internal representations^9^. The subjects’ overall correctness rates (CR) varied from 75.5 to 100% (M = 95.1%, SD = 6.4%). Based on the correction rate, we excluded two subjects with CR of 75.5% and 80.0% as they exceeded the 90th percentile of CR distribution in the group. As a result, we proceeded with 18 subjects (M = 97.07%, SD = 2.6%). In this work, we focused on the effect of the previous stimulus. To ensure that the subject accurately processed the previous stimulus, we excluded all trials with erroneous responses to previous stimuli. The amount of excluded trials varied from 1.4 to 18.9% (M = 9.8%, SD = 4.2%) of the initial number of trials.

### Statistical analysis

We performed the group-level statistics for the median RT and the CR for the current cube depending on the four different factors: current ambiguity, current orientation, previous ambiguity, previous orientation. We also contrasted the median presentation time (PT) across the same conditions to control the possible bias of presentation time. The main effects were evaluated via repeated-measures ANOVA. The post hoc analysis used either paired samples *t*-test or Wilcoxon signed-rank test, depending on the samples’ normality. Normality was tested via the Shapiro-Wilk test. We performed a statistical analysis using SPSS.

We analyzed NWP in the frequency band of 1–40 Hz in the three time-intervals of interest (TOI) (Fig. 1C). The first TOI was a 2 s interval time-locked to the current stimulus onset and included 1.5 s prestimulus and 0.5 s post-stimulus segments. The second TOI reflected a 0.5 s interval preceded the behavioral response to the current stimulus. Finally, the third TOI reflected a 1.5 s period followed the behavioral response to the previously presented stimulus.

To contrast the sensor-level NWP we used paired *t*-test in conjunction with the nonparametric cluster-based correction for the multiple comparisons and the Monte-Carlo randomization. Cluster formation required at least two neighboring sensors. A cluster was significant when the *p* value was below 0.05, corresponding to a false alarm rate of 0.05 in a two-tailed test. The number of permutations was 1000.

The source power was also contrasted via a paired *t*-test with the nonparametric cluster-based correction for the multiple comparisons. A cluster was significant when the *p* value was below 0.05, corresponding to a false alarm rate of 0.05 in a two-tailed test. The number of permutations was 2000. We performed sensor-level and source-level statistical analysis in the Fieldtrip toolbox for MATLAB.

### Source localization

We analyzed the source power in the predefined time-frequency domain chosen based on the sensor-level analysis. Using the exact low-resolution brain electromagnetic tomography (eLORETA) method, we solved the inverse problem and reconstructed source activity from the EEG at each of the predefined points over brain volume^18–20^. “Colin27” brain MRI averaged template^21^ was used to develop a boundary element method (BEM) head model with three layers (brain, skull, and scalp)^22,23^ and source space consisting of 11865 voxels inside the brain. We fitted the EEG electrodes’ positions to the template head shape. First, we re-referenced EEG signals to the common average, demeaned them, and filtered by the fourth-order Butterworth bandpass filter with the passband $f_L$ and $f_H$ representing the frequencies of interest. Then, we performed time-lock averaging across the trials in each condition and computed the covariance matrix. Finally, we normalized the resulted source power *P* in each condition to the 40-s baseline EEG recorded at the beginning of the experiment as $(P-P_{baseline})/P_{baseline}$. To match the sources’ locations with the brain’s anatomical regions, we used the Automated Anatomical Labeling (AAL) brain atlas^24^. All operations were performed using the Fieldtrip toolbox in MATLAB^25^.

## Results

**Figure 2:**
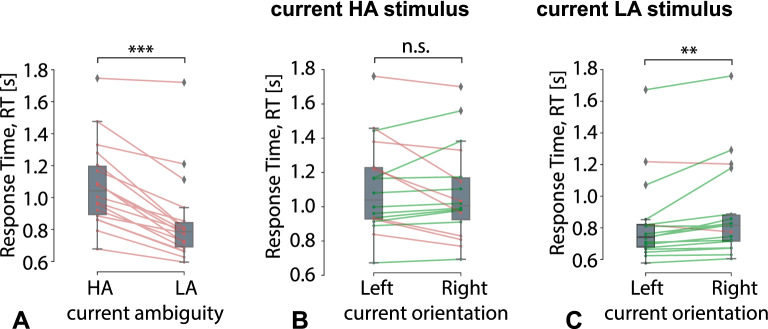
Effect of the current stimulus orientation and ambiguity on the RT, regardless of the previous stimulus orientation and ambiguity. Box-plot illustrates the median RT in the group of subjects for (**A**) HA and LA stimuli, $$p<.001^{***}$$. (**B**) HA stimuli with left and right orientations, $$p=.3$$. (**C**) LA stimuli with left and right orientation, $$p<.01^{**}$$.

**Results of behavioral response analysis.** We found a significant main effect of the current stimulus ambiguity on the RT (Table 1). It indicated that all subjects responded to the LA stimuli (M$=.84$ s, SD$=.26$) faster than to the HA stimuli (M$=1.07$ s, SD$=.26$): $t(17)=-8.125, p<.001$ (Fig. 2A). There was a main effect of the current ambiguity on the CR (Table 2). We observed that the CR for the LA stimuli was higher than for the HA stimuli. According to the Table 3, the presentation times of the HA and LA stimuli did not differ.

A significant interaction effect of the current stimulus ambiguity and orientation on the RT (Table 1) suggested that the RT might differ between the left- and right-oriented stimuli depending on their ambiguity. The post-hoc analysis demonstrated that the RT remains similar for the left-oriented (M$=1.1$ s, SD$=.27$) and the right-oriented (M$=1.07$ s, SD$=.27$) HA stimuli: $t(17)=1.07, p=.3$ (Fig. 2B). In contrast, 16 subjects responded faster to the left-oriented LA stimuli (M$=.82$ s, SD$=.26$) than to the right-oriented ones (M$=.88$ s, SD$=.29$): $Z=-3.114, p=.002$ (Fig. 2C). The main effects of the current ambiguity and current orientation on both the CR (Table 2) and the PT (Table 3) were insignificant.

**Table 1:** Median Response time to the current stimulus, RT [s] (ANOVA Summary).

Factors	$dF_1$	$dF_2$	*F*	*p*
Current ambiguity	1	17	61.701	< .001***
Current orientation	1	17	2.527	.130
Previous ambiguity	1	17	1.751	.203
Previous orientation	1	17	.402	.535
Current ambiguity * Current orientation	1	17	4.993	.039*
Current ambiguity * Previous ambiguity	1	17	6.408	.022*
Current orientation * Previous ambiguity	1	17	1.140	.301
Current ambiguity * Current orientation * Previous ambiguity	1	17	.338	.569
Current ambiguity * Previous orientation	1	17	1.549	.230
Current orientation * Previous orientation	1	17	.034	.856
Current ambiguity * Current orientation * Previous orientation	1	17	7.853	.012*
Previous ambiguity * Previous orientation	1	17	.750	.399
Current ambiguity * Previous ambiguity * Previous orientation	1	17	.525	.478
Current orientation * Previous ambiguity * Previous orientation	1	17	.089	.769
Current ambiguity * Current orientation * Previous ambiguity * Previous orientation	1	17	2.002	.175

**Figure 3:**
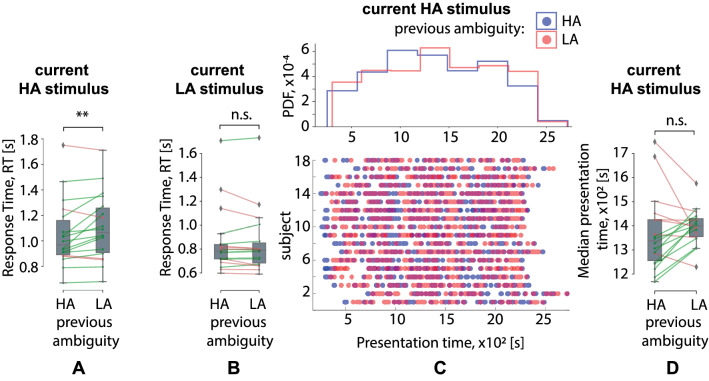
Effect of the previous stimulus ambiguity on the RT to the current stimulus. Box-plot illustrates the median RT in the group of subjects to (**A**) the HA stimuli, $p<.017^{*}$ and (**B**) LA stimuli, $p=.727$ (**B**) depending on the ambiguity of the previous stimulus. Dot-plot and histograms (**C**) show PT distributions for the HA stimuli following the HA and LA stimulus. Box-plot (D) reflects the median PT of the HA stimuli following the HA and LA stimuli in the group of subjects, $p=.355$.

Regarding the previous stimulus influence, we reported a significant interaction effect of the current and the previous stimuli ambiguity on RT (Table 1). We could interpret this interaction as the meaning that the previous stimulus ambiguity affected the RT to the current stimulus differently depending on its ambiguity. The post-hoc analysis revealed that 15 subjects responded faster to the HA stimuli if the previous stimulus was also HA (M$=1.05$ s, SD$=.26$) than for the previous LA stimuli (M$=1.1$ s, SD$=.26$): $t(17)=-2.64, p=.017$ (Fig. 3A). On the contrary, RT to the LA stimulus after the HA stimulus (M$=.85$ s, SD$=.27$) was similar as after the LA one (M$=.84$ s, SD$=.27$): $Z=-.348, p=.727$ (Fig. 3B). Besides, there was a significant interaction effect of the current ambiguity and the previous ambiguity on the CR (Table 2). At the same time, it failed to achieve significance in the post-hoc tests. Finally, the interaction effect of the current ambiguity and the previous ambiguity on the PT was insignificant (Table 3). Since the effect of the previous stimulus was central in this manuscript, we additionally considered the PT distributions of the HA stimuli followed the HA and LA stimuli (Fig. 3C) and contrasted the median PT in these conditions (Fig. 3D). We found that PT varied from 300 s to 2700 s. The median PT of HA stimuli following HA stimuli (M$=1365$ s, SD$=157$) did not differ from the median PT of HA stimuli following LA stimuli (M$=1395$ s, SD$=74$): $t(17)=-.975, p=.355$ (Fig. 3D). According to the Fig. 3D, the differences between some PTs pairs in HA vs. LA conditions are extremely large. To make the RT analysis in these conditions more convincing, we applied a strict criterion to the PT difference. Setting the threshold for the absolute PT difference ($\Delta$PT) as $2.5\times 10^2$ s, we excluded one subject with $\Delta \mathrm {PT}= 3.19\times 10^2$ s from the consideration. The RT difference in the rest of the subjects remained significant: $t(17)=2.56, p=.021$.

**Table 2:** Percentage of correct responses on the current stimulus, CR [%] (ANOVA Summary).

Factors	$dF_1$	$dF_2$	*F*	*p*
Current ambiguity	1	17	12.379	.003**
Current orientation	1	17	.618	.443
Previous ambiguity	1	17	1.788	.199
Previous orientation	1	17	.386	.543
Current ambiguity * Current orientation	1	17	1.455	.244
Current ambiguity * Previous ambiguity	1	17	6.682	.019*
Current orientation * Previous ambiguity	1	17	4.491	.049*
Current ambiguity * Current orientation * Previous ambiguity	1	17	2.723	.117
Current ambiguity * Previous orientation	1	17	.718	.409
Current orientation * Previous orientation	1	17	2.992	.102
Current ambiguity * Current orientation * Previous orientation	1	17	2.992	.399
Previous ambiguity * Previous orientation	1	17	.076	.786
Current ambiguity * Previous ambiguity * Previous orientation	1	17	.105	.750
Current orientation * Previous ambiguity * Previous orientation	1	17	2.608	.125
Current ambiguity * Current orientation * Previous ambiguity * Previous orientation	1	17	4.111	.059

**Figure 4:**
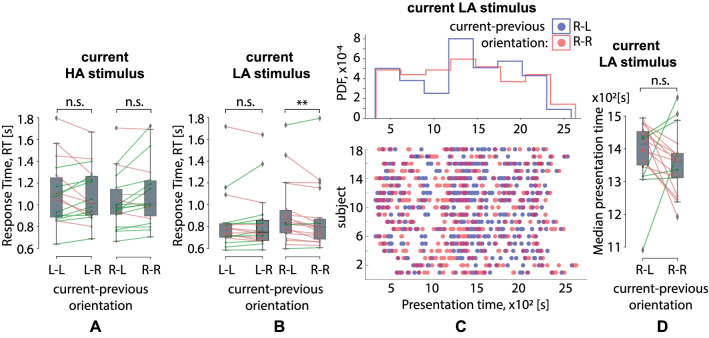
Effect of the previous stimulus orientation on RT to the current stimulus. Box-plot (**A**) illustrates median RT in the group of subjects to the left-oriented (L) and the right-oriented (R) HA stimuli depending on the orientation of the previous stimulus ($$p>0.05$$, ANOVA). Box-plot (**B**) illustrates median RT for the left-oriented and the right-oriented LA stimuli depending on the previous stimulus’s orientation $$p=.005^{**}$$. Dot-plot and histograms (**C**) show distributions of the presentation times for the right-oriented LA stimuli preceded by right-oriented (R-R) and left-oriented (R-L) stimuli. Box-plot (D) reflects the median presentation times of the right-oriented LA stimuli preceded by the right-oriented and the left-oriented stimuli, $$p=.337$$.

Finally, there was a significant interaction effect of the current ambiguity, current orientation, and the previous orientation on the RT (Table 1). Within the post-hoc analysis, we considered RT separately for HA and LA current stimuli via a repeated-measures ANOVA with the current and previous orientation taken as within-subject factors. For the HA stimuli, neither current nor previous orientations had a significant effect on the RT (Fig. 4A). For the LA stimuli, there was a significant interaction effect of the previous and current orientation on the RT: $F(1,17)=7.262, p=.015$. The post-hoc analysis revealed that the RT did not depend on the previous stimulus orientation for the left-oriented LA stimuli. In contrast, 16 subjects responded faster to the right-oriented LA stimuli if the previous stimulus was also right-oriented (M$=.87$ s, SD$=.29$) rather than after the left-oriented previous stimulus (M$=.92$ s, SD$=.29$): $Z=-2.809, p=.005$ (Fig. 4B). To exclude the possible effect of the PT bias between the conditions, we considered the PT distributions of the right-oriented LA stimuli followed the left-oriented (R-L) and the right-oriented (R-R) stimuli (Fig. 4C) and contrasted the median PT in these conditions (Fig. 4D). We found that PT varied from $3\times 10^2$ s to $27\times 10^2$ s. The median PT of the right-oriented LA stimuli followed the left-oriented stimuli (R-L) (M$=1389$ s, SD$=95$) did not differ from the median PT of the right-oriented LA stimuli followed the right-oriented stimuli (R-R) (M$=1356$ s, SD$=95$): $t(17)=-.988, p=.337$ (Fig. 4D). According to the Fig. 4D, the differences between some PTs pairs in R-R vs. R-L conditions are also large. Setting the threshold for the absolute PT difference ($\Delta$PT) as $2.5\times 10^2$ s, we excluded two subjects with $\Delta \mathrm {PT}=2.63\times 10^2$ s and $\Delta \mathrm {PT}=\Delta 2.95\times 10^2$ s from the consideration. The RT difference in the rest 16 subjects remained significant: $t(17)=2.29, p=.037$.

**Table 3:** Median presentation time of the current stimulus, PT [c] (ANOVA Summary).

Factors	$dF_1$	$dF_2$	*F*	*p*
Current ambiguity	1	17	2.1	.165
Current orientation	1	17	1.7	.21
Previous ambiguity	1	17	1.079	.313
Previous orientation	1	17	.474	.5
Current ambiguity * Current orientation	1	17	.868	.364
Current ambiguity * Previous ambiguity	1	17	.507	.486
Current orientation * Previous ambiguity	1	17	.037	.849
Current ambiguity * Current orientation * Previous ambiguity	1	17	.037	.468
Current ambiguity * Previous orientation	1	17	.1	.921
Current orientation * Previous orientation	1	17	.443	.515
Current ambiguity * Current orientation * Previous orientation	1	17	3.222	.09
Previous ambiguity * Previous orientation	1	17	.215	.649
Current ambiguity * Previous ambiguity * Previous orientation	1	17	1.63	.219
Current orientation * Previous ambiguity * Previous orientation	1	17	.154	.7
Current ambiguity * Current orientation * Previous ambiguity * Previous orientation	1	17	4.111	0.002**

### Results of EEG signals analysis

Behavioral analysis indicated that the previous stimulus affected the current stimulus processing in two cases: i) subjects responded faster to the HA stimuli following the HA stimuli; ii) subjects responded faster to the right-oriented LA stimuli following the right-oriented stimuli. We analyzed changes in neural activity that might underly these behavioral effects in three TOI (see Fig. 1C).

**Figure 5:**
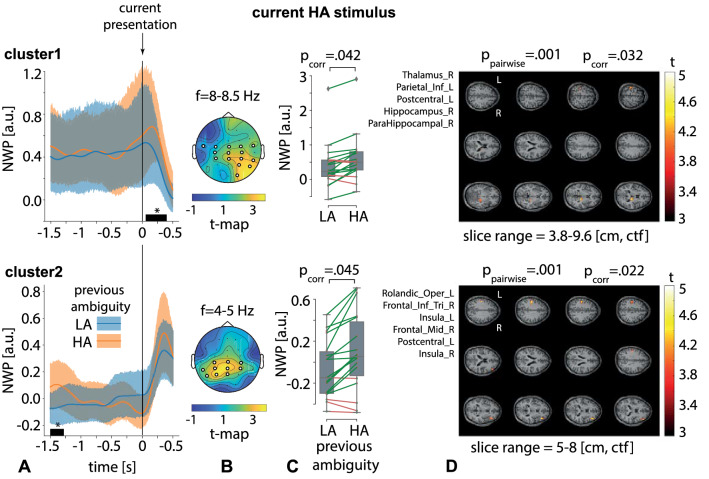
Two significant clusters as the result of NWP comparison during HA stimuli processing following the HA and LA stimuli on the 2 s interval time-locked to stimulus onset (vertical solid line). The blue and orange curves (**A**) show evolution of the NWP (group means and 95% CI) in these clusters during HA stimuli processing following the LA and HA stimuli. The scalp topogram (**B**) reflects *t*-value, and the channels demonstrating a significant change of NWP between analyzed conditions on the EEG sensor level. The box-plot (**C**) illustrates NWP in this cluster averaged over the time interval, frequency band, and channels. The slice-plot (**D**) reflects the *t*-value for the voxels demonstrating a significant change of the source power. $p_{corr}$ is corrected for multiple comparisons via the cluster-based test with the Monte-Carlo randomization technique.

First, we contrasted neural activity during processing HA stimuli following HA and LA stimuli. Contrasting the normalized wavelet power (NWP) on a 2-s interval time-locked to the current stimulus onset, including 1.5-s prestimulus and 0.5-s post-stimulus segments (TOI1 in Fig. 1C), we found two significant positive clusters.

The first sensor-level cluster with $p=.042$ appeared from .09 s to .37 s post-stimulus onset in the frequency range of $8-8.5$ Hz (Fig. 5A, top plot) and included EEG sensors {O2, P4, P8, CP3, CPz, CP4, TP8, C3, Cz, C4, FT7, FC3, FCz, FC4, FT8} in the right-lateralized occipito-parietal and bilateral central EEG rows (Fig. 5B, top plot). The averaged NWP in this cluster was higher when the HA stimulus followed the HA stimulus in 14 subjects (Fig. 5C, top plot). For the time-frequency range of this cluster, we calculated source power and contrasted it between the conditions. As a result, we observed a significant positive cluster with $p=.032$. This source-level cluster included the right part of the thalamus, left inferior parietal lobule, left postcentral gyrus, right part of the hippocampus, and right parahippocampal gyrus (Fig. 5D, top plot). The second sensor-level cluster with $p=.045$ appeared from 1.5 s to 1.28 s before stimulus onset in the frequency range of $4-5$ Hz (Fig. 5A, bottom plot) and included EEG sensors {P7, P3, Pz, TP7, CP3, CPz, CP4, C3, Cz, C4} mostly in the parietal and central EEG rows (Fig. 5B, bottom plot). The averaged NWP in this cluster was higher when HA stimulus followed HA stimulus in 14 subjects (Fig. 5C, bottom plot). We observed a significant positive cluster on the source-level with $p=.022$, that included left rolandic operculum, left inferior frontal gyrus, left and right insula, right middle frontal gyrus, and left postcentral gyrus (Fig. 5D, bottom plot).

Contrasting the NWP on a 0.5 s interval that preceded the behavioral response to the current stimulus (TOI2 in Fig. 1C), we found no significant clusters.

**Figure 6:**
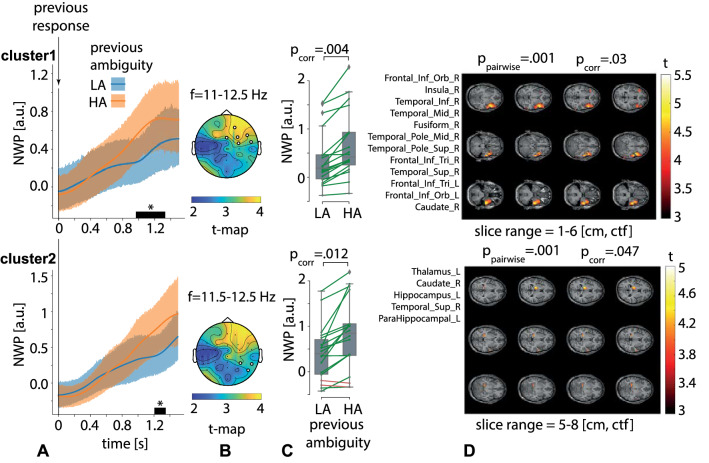
Two significant clusters as the result of NWP comparison after responding to HA and LA stimuli on the 1.5-s interval time-locked to the behavioral response (vertical solid line). The orange and blue curves (**A**) show the NWP evolution (group means and 95% CI) in these clusters after responding to LA and HA stimuli. The topogram (**B**) reflects the *t*-value and the channels demonstrating a significant change of NWP between analyzed conditions on the EEG sensor level. The box-plot (**C**) illustrates NWP in this cluster averaged over the time interval, frequency band, and channels. The slice-plot (**D**) reflects the *t*-value for the voxels included in the source-level cluster. $p_{corr}$ is corrected for multiple comparisons via the cluster-based test with the Monte-Carlo randomization technique.

Contrasting the NWP on a 1.5 s period followed the behavioral response to the previously presented stimulus (TOI3 in Fig. 1C), we found two significant positive clusters on the sensor level. The sensor-level first cluster with $p=.004$ appeared from 1.0 to 1.33 s after the behavioral response in the frequency-range of 11–12.5 Hz (Fig. 6A, top plot). It included EEG sensors {Fz, Fp2, F4, F8, FC4, FT8} in the right-lateralized frontal EEG rows (Fig. 6B, top plot). The averaged NWP in this cluster was higher after responding to the HA stimulus in all subjects (Fig. 6C, top plot). We observed a significant positive cluster ($p=.03$) on the source-level in this time-frequency range, including the bilateral frontal gyri, right temporal gyri, right insula, fusiform gyrus, and caudate nucleus (Fig. 6D, top plot). The second sensor-level cluster with $p=.012$ appeared from 1.0 to 1.1 s after the behavioral response in the frequency range of 11.5–12.5 Hz (Fig. 6A, bottom plot). It included EEG sensors {P8, CP4, TP8} in the right-lateralized temporal EEG rows (Fig. 6B, bottom plot). The averaged NWP in this cluster was higher after processing HA stimulus in 16 subjects (Fig. 6C, bottom plot). On the source level, it corresponded to a positive cluster with $p=.047$, including the left parts of the thalamus, hippocampus, and the parahippocampal gyrus, as well as the right superior temporal gyrus, and right caudate nucleus (Fig. 6D, bottom plot).

Second, we contrasted neural activity during the right-oriented LA stimulus processing, following the right- and left-oriented stimuli. Contrasting the NWP on TOI1 we found no significant clusters.

**Figure 7:**
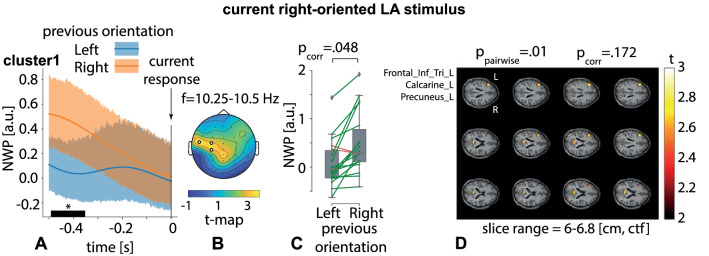
Significant cluster as the result of NWP comparison during the right-oriented LA stimuli processing following the right- and left-oriented stimuli on the 0.5-s interval time-locked to the behavioral response (vertical solid line). The orange and blue curves (**A**) show NWP evolution (group means and 95% CI) in this cluster during the right-oriented LA stimuli processing following the left-oriented and right-oriented stimuli. The topogram (**B**) reflects the *t*-value and the channels demonstrating a significant change of NWP between analyzed conditions on the EEG sensor level. The box-plot (**C**) illustrates NWP in this cluster averaged over the time interval, frequency band, and channels. The slice-plot (**D**) reflects the *t*-value for the voxels demonstrating a significant change of the source power. $p_{corr}$ is corrected for multiple comparisons via the cluster-based test with the Monte-Carlo randomization technique.

Contrasting the NWP on TOI2, we found one significant positive cluster with $p=.048$. This sensor-level cluster appeared from .49 to .37 s before the behavioral response in the frequency range of 10.25–10.5 Hz (Fig. 7A). It included EEG sensors {C3, FT7, FC3} in the left-lateralized central, temporal and frontal EEG rows (Fig. 7B). The averaged NWP in this cluster was higher when the right-oriented LA stimulus followed the right-oriented stimuli in 16 subjects (Fig. 7C). For the time-frequency range of this cluster, we calculated source power and contrasted it between the conditions. Setting the $p_{pairwise}=.001$, we did not found the source-level clusters. For the $p_{pairwise}=.01$ we obtained a positive cluster with $p=.172$ (Fig. 7D). This source-level cluster included the left inferior frontal gyrus, left calcarine sulcus, and the left precuneus. These source-level results showed potentially interesting tendencies without being supported by the cluster-level statistics. To quantify the change of source power in these zones, we computed Cohen’s *d* and counted the number of subjects demonstrating the effect. As a result, we found a medium effect for the left inferior frontal gyrus ($d=0.55$, 14 subjects), the left precuneus ($d=0.49$, 15 subjects) and the left calcarine sulcus ($d=0.52$, 15 subjects).

**Figure 8:**
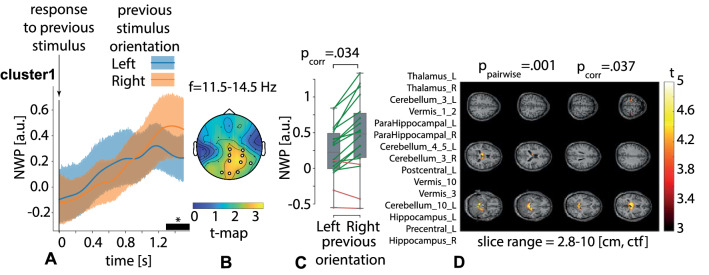
Significant cluster as the result of NWP comparison after responding to the right- and left-oriented stimuli on the 1.5-s interval time-locked to the behavioral response (vertical solid line). The orange and blue curves (**A**) show the NWP evolution (group means and 95% CI) in these clusters after responding to the left- and right-oriented stimuli. The topogram (**B**) reflects the *t*-value and the channels demonstrating a significant change of NWP between analyzed conditions on the EEG sensor level. The box-plot (**C**) illustrates NWP in this cluster averaged over the time interval, frequency band, and channels. The slice-plot (**D**) reflects the *t*-value for the voxels included in the source-level cluster. $p_{corr}$ is corrected for multiple comparisons via the cluster-based test with the Monte-Carlo randomization technique.

Contrasting NWP on TOI3, we found one significant positive cluster with $p=.034$ in the frequency range of 11.5–14.5 Hz on the time interval of 1.3–1.5 s after the behavioral response (Fig. 8A). It included EEG sensors {Pz, P4, CPz, CP4, Cz, C4} in the right-lateralized occipito-parietal and central EEG rows (Fig. 8B). The averaged NWP in this cluster was higher after responding to the right-oriented stimulus in 15 subjects (Fig. 8C). On the source level, it corresponded to the significant positive cluster with $p=.037$, including left and right parts of the thalamus, hippocampus, parahippocampal gyrus, left precentral and postcentral gyri, cerebellum, and vermis (Fig. 8D).

## Discussion

We revealed that the subjects responded to LA stimuli faster and more correctly than to HA stimuli that coincides with our previous findings^12,26^. In the latter case, they deal with ambiguous sensory information and may increasingly rely on the top-down processes, such as expectations and memory, to make a correct decision^9,13^. When processing LA stimuli, subjects responded quicker to the left-oriented stimuli than to the right-oriented ones. We suppose that the Necker cube has high spatial dimensions and covers both visual fields. The cube’s morphology suggested that the sensory evidence for the left orientation appeared mostly in its left part. When visually examining stimulus from left to right, the observer starts seeing the internal edges that provide additional information about the right orientation. There is a view that subjects respond faster to the stimuli in the left visual field due to the right-lateralization of the attentional networks^27^. Thus, we suppose that participants quickly find the signs of left-orientation in the left visual field. Future studies should validate this hypothesis by using eye-tracking systems.

Regarding the previous stimulus effect, we observed higher speed and correctness of the response to HA stimulus folowing HA stimulus. Moreover, we reported a faster response to the right-oriented LA stimulus following the right-oriented stimulus. We hypothesize that these two behavioral effects result from the different mechanisms of brain activity. In the first case, we suppose that previous stimulus modulates attentional state and mobilizes neural networks used for ambiguous stimuli processing. It improves the processing of ongoing HA stimulus, contributing to its disambiguation and orientation determination. In the second case, we suppose that the brain learns the signs of the right orientation. It facilitates orientation determination of the ongoing right-oriented stimulus by matching its orientation with the previously learned one.

Analysis of the prestimulus and post-stimulus neural activity provided evidence supporting our hypothesis. We observed high prestimulus theta-band power when HA stimulus followed HA stimulus. The source analysis revealed increased activation of the right and the left insula, right frontal cortex, and the left postcentral cortex. Activity in the theta-band appears to coordinate neural activity in the remote regions^28^, including neural interactions in the fronto-parietal attentional networks^29^. The right insula and right frontal cortex formed the core of a ventral attentional network^30^. Activity in these areas, together with the parietal cortex, increases during visual tasks. The right insula and right inferior frontal cortex are also among the most critical prefrontal regions for cognitive control, enabling detecting behaviorally salient stimuli^31^. Finally, the insular cortex plays a crucial role in accumulating evidence to process uncertainty in the context of decision-making^32^. Taken together, it indicates activation of the neural networks related to attention and uncertainty processing before HA stimulus onset.

Processing of the HA stimulus following the HA stimulus also induced high theta-band power for 0.1–0.35 s post-stimulus onset. The source analysis revealed increased activation of the thalamus, hippocampus, parahippocampal gyrus, and the left parietal and postcentral gyri. Traditionally, the stimulus-related theta-band power correlates with memory retrieval and decision making. During retrieval, the power of left-parietal theta oscillations increased in proportion to how well a test item was remembered^33^. Finally, thalamic theta-band power also increased during retrieval^34^. The right-lateralized hippocampal activation is essential to retrieving the sensory-based memory details^35^. While the Ref. ^35^ relates hippocampal activation with the long-term memory, its activity appears to subserve encoding and retrieval in a working memory paradigm^36,37^. These results may suggest that the processing of HA stimulus utilizes the information retrieved from memory. Increased parahippocampal activity may also confirm this view. This area exhibits stronger activation when processing old stimulus configurations than new configurations^38^.

Improved response time to the right-oriented LA stimulus following the right-oriented stimulus did not accompany changes of the prestimulus and post-stimulus neural activity. Simultaneously, we observed activation of the precuneus gyrus and the left inferior frontal gyrus before the behavioral response. Previously, Lundstrom and colleagues suggested that both the precuneus and the left inferior PFC were essential for the regeneration of episodic contextual associations and that they activated together during correct source retrieval^39,40^. The absence of differences in the post-stimulus period may indicate that retrieval-related increase of power attenuates due to repetition suppression. Repetition suppression means that the stimulus is the same as a previous event, with minimal encoding need, and usually reduces the medial temporal lobe activation, including hippocampus^41^.

Together, these results may suggest that stimulus processing partly relies on the matching information between the current and previously presented stimuli. Increased hippocampal activation during the earlier (0.1–0.3 s) stage of HA stimulus processing may evidence retrieving information to disambiguate it and define orientation. Increased activation of the precuneus and the left prefrontal cortex before responding to the right-oriented LA stimulus following the right-oriented stimulus may indicate a match between the orientations.

Supposing that the brain retrieves the previous stimulus’s features to process the next stimulus, we inspected intervals between stimuli to find neural signs of the encoding process. Earlier, Ben-Yakov and Dudai reported that the hippocampal activation immediately following event offset plays a role in encoding the episode into memory and can predict subsequent memory for it^42^. Similarly, we observed increased hippocampal activation for $\sim 1.5$ s after responding to the HA stimuli and the right-oriented LA stimuli. Combining these findings with the behavioral data, we assume that the interpretation of HA stimulus may require more information than the interpretation of LA stimulus. Similarly, the interpretation of the right-oriented stimulus requires more information than the interpretation of the left-oriented one. After the stimulus offset, the percept-related and the decision-related stimulus information is encoded in memory. Enhanced hippocampal activation may reflect the amount of encoded information.

Literature usually associates hippocampal activation and the activation of the precuneus and the left prefrontal cortex with the long-term episodic memory and stimulus-stimulus associations^43^. Simultaneously, the hippocampus subserves the short-term associative memory^44^. Associative memory refers to the ability to remember relationships between two or more items or between an item and its context, e.g., between the pairs of pictures^41,43^ or between the pictures and words^45^. Grön et al. also reported that hippocampal activation during a response to the complex geometric patterns might reflect encoding and retrieval of intra-image associations^46^. Thus, participants may learn the associations between the Necker cube features in the most demanding cases, such as HA and the right-oriented LA stimuli. After the offset of these stimuli, these associations are encoded into the memory. When the previously seen stimulus is reexperienced, the sensory information is matched to stored memory representations, improving the behavioral response. We suppose that there is a difference between the HA and the right-oriented LA stimuli. In the first case, the brain encodes stimulus details to use them for defining the orientation. In the latter case, it learns the signs of the particular (right) orientation. Therefore, we observe further thalamic activation during HA stimulus processing. The absence of such activation during the right-oriented LA stimulus processing may reflect that the stimulus orientation matches the previously seen stimulus’s orientation. Moreover, we suppose that the HA stimulus modulates the attentional network’s activity, including right-lateralized frontal and temporal cortices, improving the next HA stimulus’s processing.

Finally, this study has limitations. While a high-density EEG enables detecting deep subcortical activity^47^, we used a small number of EEG channels, and an averaged MRI, which may not be sufficient for reliable localization of the reported neuronal activity in source space. Therefore, we did not report the lateralization of the hippocampal activity, which is also essential for memory encoding and retrieval.
